# Hsa_Circ_0007843 Acts as a mIR-518c-5p Sponge to Regulate the Migration and Invasion of Colon Cancer SW480 Cells

**DOI:** 10.3389/fgene.2020.00009

**Published:** 2020-02-25

**Authors:** Jin Hua He, Ze Ping Han, Jin Gen Luo, Jian Wei Jiang, Jia Bin Zhou, Wei Ming Chen, Yu Bing Lv, Meng Ling He, Lei Zheng, Yu Guang Li, Ji Dong Zuo

**Affiliations:** ^1^ Department of Laboratory Medicine, Central Hospital of Panyu District, Guangzhou, China; ^2^ Digesting Center, Central Hospital of Panyu District, Guangzhou, China; ^3^ Department of Biochemistry, Medical College, Jinan University, Guangzhou, China; ^4^ Department of Laboratory Medicine, Nanfang Hospital, Southern Medical University, Guangzhou, China; ^5^ Department of Gastrointestinal Surgery, The First Affiliated Hospital, Sun Yat-sen University, Guangzhou, China

**Keywords:** hsa_circ_0007843, matrix metallopeptidase 2, miR-518c-5p, colon cancer, invasion, migration

## Abstract

Circular RNA (circRNA), a type of RNA that is widely expressed in mammalian cells, is considered to be essential in tumorigenesis. CircRNA can regulate target gene expression by interacting with the corresponding microRNA (miRNA). Our preliminary results showed that the expression levels of 1,817 circRNAs were significantly different in colon cancer tissue compared with paracancerous tissue, of which 1,236 were upregulated and 581 were downregulated. By using RT-PCR, we confirmed that the expression of hsa_circ_0007843, hsa_circ_0010575, hsa_circ_0007331, and hsa_circ_0001615 was significantly higher in colon cancer tissue than in normal colonic tissue; however, the expression levels of hsa_circ_0014879 and hsa_circRNA_401801 were not significantly different between normal and neoplastic colonic tissue. Among the circRNAs that were confirmed to be upregulated in colon cancer tissue, hsa_circ_0007843 was also found to be highly expressed in colon cancer SW480 cells. Overexpression of hsa_circ_0007843 promoted the invasion and migration of SW480 cells, whereas its downregulation suppressed their invasion and migration. Overexpression of hsa_circ_0007843 promoted tumor growth, whereas its downregulation inhibited tumor growth. We found that hsa_circ_0007843 interacted with miR-518c-5p and suppressed its expression, and miR-518c-5p interacted with matrix metallopeptidase 2 (MMP2) and promoted its expression and translation. Taken together, this study demonstrated that hsa_circ_0007843 acted as an miRNA sponge to regulate MMP2 expression by removing the inhibitory effect of miR-518c-5p on MMP2 gene translation, which further affected the invasive capability of SW480 cells.

## Introduction 

Colon cancer, a common gastrointestinal tumor, remains a serious threat to human health with the 3rd highest morbidity and mortality rate worldwide (Siegel et al., 2017). The development of colon cancer is a multistep process in which the abnormal expression of genes plays an important role (Qi and Ding, 2018). Although colon cancer has been studied widely, its pathogenesis is not fully understood. Hence, in-depth studies are required for the further elucidation of the molecular mechanism involved in the carcinogenesis of human colon cancer, which will provide new insights for the diagnosis, prognosis, and treatment of colon cancer.

Circular RNA (circRNA), a newly discovered non-coding RNA, has become a research hotspot in relevant fields. Unlike a linear RNA molecule that contains a 5′ cap and a 3′ polyadenylation tail, circRNA forms a covalently closed continuous loop and therefore does not have 5′ and 3′ ends (Memczak et al., 2013; Wang et al., 2018). CircRNAs are largely expressed in the eukaryotic transcriptome and participate in the regulation of gene expression (Guttman and Rinn, 2012; Holdt et al., 2018) and can therefore be considered essential in tumorigenesis (Arnaiz et al., 2018).

CircRNAs can regulate target gene expression by interacting with corresponding microRNAs (miRNAs) (Zhang et al., 2015). CircRNAs can function as miRNA “sponges” and regulate gene transcription (Hansen et al., 2013; Sang et al., 2018). For example, circRNA-ITCH increases ITCH gene expression by acting as an miRNA sponge by interacting with miR-7, miR-17, and miR-214. Overexpression of ITCH promotes the ubiquitination and degradation of phosphorylated Dv12 protein, thereby inhibiting the Wnt signaling pathway and suppressing the progression of cancer (Li et al., 2015). circRNA-000284 affects proliferation and invasion of cervical cancer cell *via* sponging miR-506 (Ma et al., 2018).

In the present study, we retrospectively collected colon cancer tissue samples from newly diagnosed patients. By using microarray analysis, we examined the circRNA expression profile of colon cancer tissue. CircRNAs whose expression levels were confirmed to be altered in colon cancer tissue were subjected to further *in vitro* analysis. By studying the biological function of the identified circRNAs and the molecular mechanism involved, we hope to provide invaluable information for the diagnosis, prognosis, and treatment of colon cancer.

## Materials and Methods

### Sample Collection

Clinical tissue specimens from patients newly diagnosed with colon cancer were obtained from the archives of the Central Hospital of Panyu District with informed consent from the patients and with the approval of the institutional Ethics Committee (Guangdong, China). In this study, we collected tumor tissue and paired normal adjacent tissue from 30 patients with colon cancer. All of the samples were collected at the General Surgery Department of Panyu Central Hospital of Guangzhou from 2013 to 2018. The patients ranged in age from 45 to 82 years, with an average age of 65.66 ± 12.14 years (20 men, average age 61.8 ± 10.39 years; 10 women, average age 65.6 ± 13.69 years). None of the patients received radiotherapy or chemotherapy before surgical resection of the pathological tissue. All of the tissue samples were immediately snap-frozen in liquid nitrogen after surgical excision and stored at −80°C until total RNA was extracted for further experimentation. Basic information of the 30 selected subjects selected in this study is shown in **Table 1**.

**Table 1:** The bascial data analysis of the 30 patients.

Indicator	
Male (n)	20
Female (n)	10
Mean age of male (years)	61.8 ± 10.39
Mean age of female (years)	65.6 ± 13.69
TNM stage	
(I/II/III/IV )	6/1/2/18/3
Tumor volume (<5 cm) (n)	12
Tumor volume (>5 cm) (n)	18

### Cell Culture

293T, FIHC, HCT-116, HT-29, SW480, LOVO, and SW620 cell lines were purchased from Yu Jia Bio Technology Co. (Guangzhou, China). The cell lines were cultured in Dulbecco’s Modified Eagle’s Medium/Nutrient Mixture F-12 Ham (DMEM/F12) containing 10% fetal bovine serum and were maintained in a humidified incubator at 37°C with 5% CO_2_. The medium was changed every other day, and the cells were grown to 80–90% confluence and digested with 0.25% trypsin and sub-cultured. Cells in logarithmic growth with 95% viability were subjected to further experimentation.

### CircRNA Microarray Analysis

Total RNA was extracted from the colon cancer tissue samples and paracancerous tissues (separated by a margin of 5 cm) with the TRIzol reagent (Invitrogen, Carlsbad, CA) according to the manufacturer’s instruction.

The tissue samples were sent to KangChen Bio-tech Inc. (Shanghai, China) for circRNA microarray analysis using a human 8×15K circRNA array (Arraystar Inc., Rockville, MD), which contains 9114 circRNA probes. Each circRNA was identified by using a specific probe that targets the specific splice junction of circRNA. Sample labeling and array hybridization were performed according to the manufacturer’s protocol (Arraystar, Inc.). The R software package (R version 3.1.2) was used to normalize the raw data and subsequent data processing. Two groups of profile differences (tumor samples vs. paracancerous tissue samples) and the absolute fold change for each circRNA were computed.

### Quantitative Real-Time RT-PCR

M-MLV reverse transcriptase was used for the reverse transcription of mRNA to cDNA, which was later used as the PCR template. Each experiment was performed in triplicate. The expression levels of U6 and β-actin were used as an internal control for mRNAs. The primers used in quantitative real-time PCR analysis are shown in **Table 2**.

**Table 2:** The primer for gene.

Gene	The sequence for primer	Length (bp)
β-actin(H)	F:5′-GTGGCCGAGGACTTTGATTG-3′; R:5′;-CCTGTAACAACGCATCTCATATT-3′;	73
hsa_circ_0007843	F:5′;-TCCGAAGATGGCTGAATGTG-3′; R:5′;-TCCCAATCAGGCCGCTCT-3′;	151
hsa_circ_0010575	F:5′;-CTGCCATCCAGGTGTGAA-3′; R:5′;-AGTCGTGGACGAGGAAGC-3′;	137
hsa_circ_401801	F:5′;-CAGTTTGCTGTTCATGGAGAC-3′; R:5′;-GGTGGGGACTGGTGCTAT-3′;	121
hsa_circ_0001615	F:5′;-TGATCGAACTGGCAGACG-3′; R:5′;-CTCCAGGAACACTTTGAGGA-3′;	128
hsa_circ_0007331	F:5′;-GAATGGGATTCGAGACCTG-3′; R:5′;-TTCTTCCAAAGCTGCCTGT-3′;	122
hsa_circ_0014879	F:5′;-TCTCCCTGTACGTTCTTATCTGC-3′; R:5′;-CTGCTCCCTTTGCTGGACATC-3′;	197
β-actin(H)	F:5′;-GTGGCCGAGGACTTTGATTG-3′; R:5′;-CCTGTAACAACGCATCTCATATT-3′;	73
miR-518c-5p	F:5′;-ATGGTTCGTGGGTCTCTGGAGGGAAGCACTTTC-3′; R:5′;-GTGCAGGGTCCGAGGT-3′;	89
MMP2	F:5′;-GATGCCGCCTTTAACTGG-3′; R:5′;-TCAGCAGCCTAGCCAGTCG-3′;	278
RT(miRNAs)	5′;-CTCAACTGGTGTCGTGGAGTCGGCAATTCAGTTGAGTTCCCAT-3′;	

### Cell Transfection and Grouping

The design and synthesis of small interfering RNAs (siRNAs) for hsa_circ_0007843 and matrix metallopeptidase 2 (MMP2), the construction of a lentiviral vector overexpressing miR-518c-5p, and the construction of a lentiviral vector expressing si-hsa_circ_0007843 were conducted by Sangon Biotech Co., Ltd. (Shanghai, China). A lentiviral vector overexpressing hsa_circ_0007843 was produced by Jisai Biotech Co., Ltd. (Guangzhou, China). An inhibitor for miR-518c-5p was purchased from RiboBio Co., Ltd. (Guangzhou, China). Transfection was performed using Lipofectamine 2000 (Invitrogen) according to the manufacturer’s instructions (**Table 3**). The transfected cells were divided into six groups: control group (no infection), hsa_circ_0007843 group (infected with lentiviral vector expressing hsa_circ_0007843), si_hsa_circ_0007843 group (infected with lentiviral vector expressing si_hsa_circ_0007843), miR-518c-5p group (infected with lentiviral vector overexpressing miR-518c-5p), si-MMP2 group (transfected with si- MMP2), and NC group (infected with lentiviral vector expressing negative control sequence).

**Table 3:** The siRNA sequence.

Gene	siRNA (5´- 3´)
hsa_circ_0007843	sense: GGCAGCATACAGGAAGATGAA antisense: UUCAUCUUCCUGUAUGCUGCC
Negative control sequence	sense: UUCUCCGAACGUGUCACGUUUC antisense: GAAACGUGACACGUUCGGAGAA
MMP2	Sense: ACUUUUCUCCUCUUUUUUCCU antisense: GAAAAAAGAGGAGAAAAGUGG

### Colony Formation Assay

The cells were divided into four groups: control group (no infection), hsa_circ_0007843 group (infected with lentiviral vector expressing hsa_circ_0007843), si_hsa_circ_0007843 group (infected with lentiviral vector expressing si_hsa_circ_0007843), and NC group (infected with lentiviral vector expressing negative control sequence). SW480 cells in logarithmic growth were plated at a density of 100 cells/well in a 6-well plate and incubated at 37°C with 5% CO_2_ for 1 week. The supernatant in each well was discarded and the cells were washed twice with 1× phosphate-buffered saline (PBS). The cells were fixed for 15 min in 4% paraformaldehyde and stained for 15 min with crystal violet in methanol. Upon discarding the staining solution, the plates were allowed to air-dry, and the colonies were observed under a microscope.

### Scratch-Wound Assay

The cells were divided into four groups: control group (no infection), hsa_circ_0007843 group (infected with lentiviral vector expressing hsa_circ_0007843), si_hsa_circ_0007843 group (infected with lentiviral vector expressing si_hsa_circ_0007843), and NC group (infected with lentiviral vector expressing negative control sequence). Cells in logarithmic growth were plated and allowed to grow until 100% confluence was reached. The cell monolayer was scraped in a straight line with a pipette tip to create a gap. Debris was removed by washing the cells 3 times with 1× PBS. Culture medium was added and the cells were allowed to grow for 24 and 48 h. Microscopic images were taken at 0, 24, and 48 h. For each image, the distance between the two edges of the scratch was measured using ImageJ software.

### Transwell Assay

The cells were divided into four groups: control group (no infection), hsa_circ_0007843 group (infected with lentiviral vector expressing hsa_circ_0007843), si_hsa_circ_0007843 group (infected with lentiviral vector expressing hsa_circ_0007843 siRNA), and NC group (infected with lentiviral vector expressing negative control sequence). Matrigel (Beijing Xia Si Biotechnology Co., Ltd., Beijing, China) was diluted in pre-chilled serum-free medium at a volume ratio of 1:3, and 40 μL pre-chilled serum-free medium was added into the pre-chilled transwell chamber. The Matrigel was solidified by incubation at 37°C for 2 h. The excess liquid was removed from the chamber and 100 and 600 μL serum-free medium was added to the upper and lower chamber, respectively. The plate containing the transwell chambers was incubated overnight at 37°C. On the day after cell transfection, 1.0 × 10^5^ cells were re-suspended in 100 μL serum-free DMEM-F12. The cells were added to the upper transwell chamber, and 600 μL complete medium was added to the lower chamber at the same time. After incubation at 37°C with 5% CO_2_ for 24 and 48 h, the surface cells and Matrigel in the upper chamber were removed using a cotton swab and the cells were observed under an inverted microscope. The cells at the lower surface of the upper chamber were stained with crystal violet and washed with 33% acetic acid. The absorbance of the solution was measured at 570 nm.

### *In Vivo* Treatment

The cells were divided into four groups: control group (no infection), hsa_circ_0007843 group (infected with lentiviral vector expressing hsa_circ_0007843), si_hsa_circ_0007843 group (infected with lentiviral vector expressing si-hsa_circ_0007843), and NC group (infected with lentiviral vector expressing negative control sequence). A total of 20 athymic BALB/c nude mice (weight, 18–20 g) were purchased from Guangdong Medical Laboratory Animal Center (Foshan, China; animal production license, NO: 440035458020). Five cell lines (SW480, SW480-NC, hsa_circ_0007843-SW480, and si-circ_0007843-SW480) were digested with 0.25% trypsin, washed with PBS, counted by trypan blue staining, and adjusted to a concentration of 1.0 × 10^6^ cells/mL, and 0.1-mL aliquots were used each time. After mixing with Matrigel (Beijing Xia Si Biotechnology Co., Ltd.), the cells were injected subcutaneously between the abdominal ribs of specific pathogen free-grade male nude mice aged up to 4 weeks. The tumor weight of the mice was observed.

### Luciferase Reporter Assay

The cells were divided into the following groups: control group (psiCHECK-2-cirRNA/psiCHECK-2-mRNA, psiCHECK-2-cirRNA-mut/psiCHECK-2-mRNA-mut), inhibitor group (inhibitor for miRNA + psiCHECK-2-cirRNA/psiCHECK-2-mRNA, psiCHECK-2-cirRNA-mut/psiCHECK-2-mRNA-mut), NC group (negative control sequence + psiCHECK-2-cirRNA/psiCHECK-2-mRNA or psiCHECK-2-cirRNA-mut/psiCHECK-2-mRNA-mut), NCI group (inhibitor for negative control + psiCHECK-2-cirRNA/psiCHECK-2-mRNA or psiCHECK-2-cirRNA-mut/psiCHECK-2-mRNA-mut sequence), and hsa_miR-518c-5p group (hsa_miR-518c-5p mimics + psiCHECK-2-cirRNA/psiCHECK-2-mRNA or psiCHECK-2-cirRNA-mut/psiCHECK-2-mRNA-mut). Genomic DNA was extracted from SW480 cells and used as the template for PCR, and *Xho*I and *Not*I restriction sites were introduced. The PCR amplification product was double-digested with the respective enzymes and cloned into the psiCHECK-2 vector to generate psiCHECK-2-cirRNA/psiCHECK-2-miRNA. A point mutation was introduced into the ligation product to generate the psiCHECK-2-cirRNA-mut/psiCHECK-2-miRNA-mut vector. 293T cells were transfected with miRNA mimics, negative control vector, miRNA inhibitor, psiCHECK-2-cirRNA/psiCHECK-2-miRNA vector, or psiCHECK-2-cirRNA/psiCHECK-2-miRNA-Mut vector (He et al., 2019).

A dual-luciferase assay (Promega, Madison, WI) was then performed according to the manufacturer’s instruction.

### Western Blot Analysis

The cells were divided into four groups: control group (no infection), si_hsa_circ_0007843 group (infected with lentiviral vector expressing si-hsa_circ_0007843), NC group (infected with lentiviral vector expressing negative control sequence), and miR-518c-5p group (infected with lentiviral vector overexpressing miR-518c-5p). When the cells reached 80% confluence, the supernatant was discarded and the cells were washed with pre-chilled 1× PBS. Then, 320 μL cell lysis buffer (RIPA with 3.2 µL PMSF) was added to the cells in order to extract cellular protein. After incubation for 30 min on ice, the cells were scraped into a 1.5 mL centrifuge tube and centrifuged for 15 min at 12,000 rpm, 4°C. Protein quantification was performed using a spectrophotometer (NanoDrop ND-1000; Thermo Fisher Scientific, Waltham, MA). Proteins were separated by sodium dodecyl sulfate-polyacrylamide gel electrophoresis and electrotransferred to polyvinylidene fluoride membranes. The membranes were incubated with a primary antibody against MMP2 at 4°C overnight, washed extensively with 0.1% Tween-20 in PBS, and incubated with a secondary antibody conjugated to horseradish peroxidase (1:1,000) at room temperature for 3 h. Immunolabeling was visualized using the ECL system.

### Statistical Analysis

Data are expressed as the mean ± standard deviation and analyzed using SPSS20.0 statistical software. Statistical comparisons were performed using one-way analysis of variance.

## Results

### Hsa_circ_0007843 Is Highly Expressed in Colon Cancer Tissue and SW480 Cells

**Figure 1:**
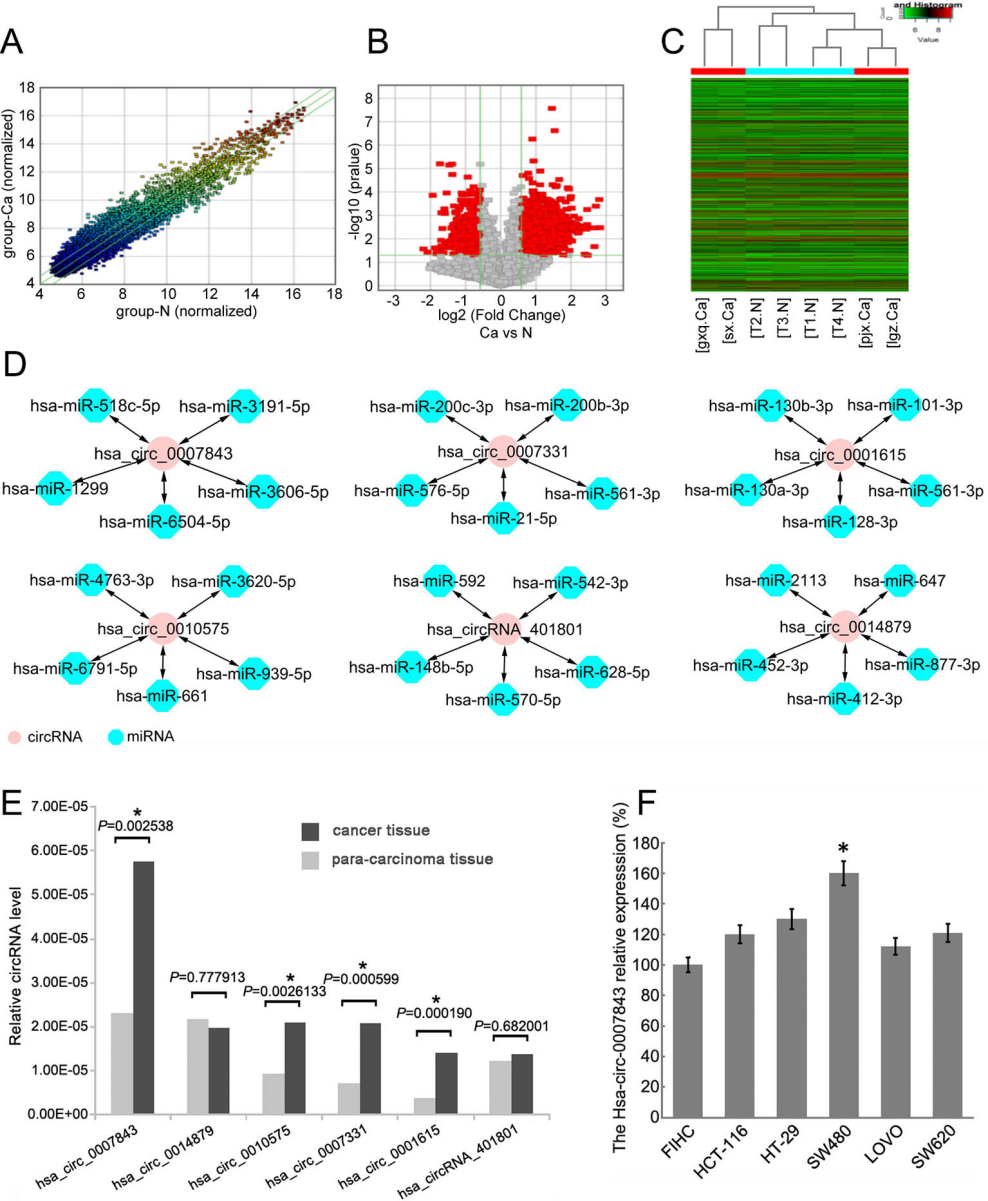
Hsa_circ_0007843 expression is upregulated in colon cancer tissue and cell lines. **(A)** Scatter-plot showing circRNA expression variation between colon cancer tissue samples and paracancerous tissue samples. The values of the X and Y axes represent the normalized signal values. The circRNAs above the top green line and below the bottom green line indicates a greater than 2.0-fold change of the circRNAs between the two compared samples. **(B)** Volcano plots showing the differential expression of circRNAs. **(C)** Hierarchical clustering heat map showing the different circRNA expression profiles of the 8 samples. **(D)** miRNA targets of the 6 circRNAs predicted by bioinformatics software. **(E)** Relative expression of the 6 circRNAs in colon cancer and paracancerous tissues. ^*^ *P* < 0.05. **(F)** Relative expression of hsa_circ_0007843 in colon cancer cell lines by RT-PCR. Each bar represents the mean of 3 independent experiments. ^*^ *P* < 0.05.

Total RNA was isolated from 4 colon cancer tissue samples and 4 paracancerous tissue samples that were separated by a margin of 5 cm. By using Arraystar circRNA microarray analysis, we identified 1817 circRNAs with clearly different expression profiles in colon cancer tissue, of which 1,236 were upregulated and 581 were downregulated (**Figures 1A–C**). Six circRNAs with the most significant change in expression were selected for subsequent study. Their corresponding chromosomal locations were analyzed using bioinformatics software. As shown in **Table 4**, most of the circRNAs corresponded to protein-coding exons. The potential miRNA targets of these circRNAs were also predicted (**Figure 1D**).

**Table 4:** Differential expression of circRNA in colon cancer and paracancerous tissues was screened by CircRNA microarray.

Regulation	circRNA	Chrom	Strand	txStart	txEnd	circRNA_type	best_transcript	GeneSymbol	*P*-value
Up	hsa_circ_0007843	chr11	–	128993340	129034322	Exonic	NM_001142685	ARHGAP32	0.000399138
Up	hsa_circ_0007331	chr3	–	195101737	195112876	Exonic	NM_012287	ACAP2	0.003367649
Up	hsa_circ_0001615	chr6	–	79752559	79770535	Exonic	NM_017934	PHIP	0.000555769
Up	hsa_circ_0010575	chr1	–	22157474	22207995	Exonic	NM_005529	HSPG2	0.002443045
Up	hsa_circ_0014879	chr1	–	160206924	160231148	Exonic	NM_015726	DCAF8	0.003808792
Up	hsa_circRNA_401801	chr10	–	14643337	14643566	Intronic	ENST00000181796	FAM107B	0.04496253

By using RT-PCR, we further verified the expression profiles of the 6 circRNAs in 26 paired colon cancer and paracancerous tissue samples The results indicated that the expression levels of hsa_circ_0007843, hsa_circ_0010575, hsa_circ_0007331, and hsa_circ_0001615 were higher in colon cancer tissue compared with normal colonic tissue (*P* < 0.05); however, the expression levels of hsa_circ_0014879 and hsa_circRNA_401801 were not different between normal and neoplastic colonic tissues (*P* > 0.05). Among the circRNAs that were confirmed to be upregulated in colon cancer tissue, hsa_circ_0007843 expression was significantly increased (**Figure 1E**). *In vitro* analysis showed that hsa_circ_0007843 was highly expressed in colon cancer SW480 cells compared to FIHC, HCT-116, HT-29, LOVO, and SW620 cells (**Figure 1F**). Hence, hsa_circ_0007843 has the potential to be utilized as a biomarker for the early diagnosis of colon cancer.

### Hsa_circ_0007843 Affects Colon Cancer SW480 Cell Colony Formation, Invasion, and Migration

**Figure 2:**
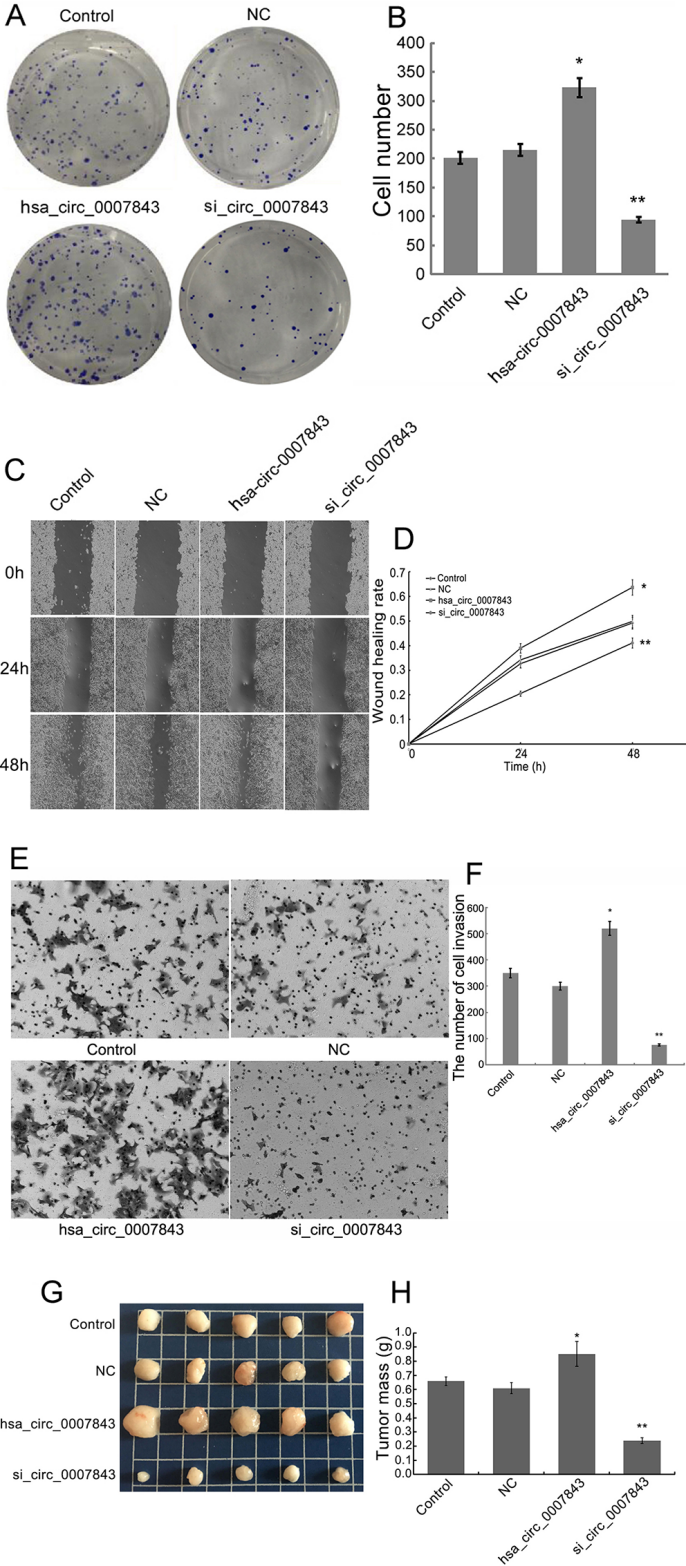
Hsa_circ_0007843 exerts oncogenic effects on colon cancer SW480 cells. **(A)** Images from the colony formation assay with different groups of SW480 cells (×40). **(B)** Bar figures representing the number of colonies formed in each group; each bar represents the mean of 3 independent experiments. **(C)** Images from the scratch-wound assay with different groups of SW480 cells (×40). **(D)** Wound healing rates for the different groups of SW480 cells; each curve represents the mean of 3 independent experiments. **(E)** Images from the transwell assay with different groups of SW480 cells (×40). **(F)** Bar figures representing the number of invaded SW480 cells in each group; each bar represents the mean of 3 independent experiments. **(G)** Representative images of tumor-bearing xenograft mice. **(H)** Tumor weight of the nude mice in each group; each bar represents the mean of 3 independent experiments. ^*^ *P* < 0.05, **P < 0.01 compared with the control and NC groups. Control (no infection), hsa_circ_0007843 (infected with lentiviral vector expressing hsa_circ_0007843), si_hsa_circ_0007843 (infected with lentiviral vector expressing hsa_circ_0007843 siRNA), and NC (infected with lentiviral vector expressing negative control sequence).

In order to clarify the effect of hsa_circ_0007843 on SW480 cells, we infected the cells with hsa_circ_0007843 siRNA and a lentiviral vector overexpressing hsa_circ_0007843. At 1 week after infection, we observed that overexpression of hsa_circ_0007843 significantly promoted SW480 cell colony formation, whereas suppressing hsa_circ_0007843 expression inhibited SW480 cell colony formation (**Figures 2A, B**). Scratch-wound and transwell assays were performed to examine the effect of hsa_circ_0007843 on SW480 cell migration and invasion. Our results showed that the migration ability of SW480 cells was significantly enhanced in the hsa_circ_0007843 group compared with the si_hsa_circ_0007843, control, and NC groups (**Figures 2C, D**). In addition, the transwell assay demonstrated that the number of invading cells was obviously increased in the hsa_circ_0007843 group compared with the si_hsa_circ_0007843 group (**Figures 2E, F**). Therefore, these results suggested that overexpressing hsa_circ_0007843 promoted colon cancer SW480 cell colony formation, invasion, and migration, while suppressing its expression inhibited SW480 cell colony formation, invasion, and migration.

### Hsa_circ_0007843 Affects Tumor Growth

SW480 tumor xenografts were established in athymic nude mice to evaluate the effects of hsa_circ_0007843 on cancer cell growth *in vivo*. Compared with the untreated animals, the application of si-hsa_circ_0007843 and hsa_circ_0007843 significantly affected tumor mass, whereas no effect was observed in the negative control group (**Figures 2G, H**). No body weight loss or diarrhea was detected and all animals (treated and untreated) survived. These results showed that reducing hsa_circ_0007843 expression effectively inhibited colon cancer growth *in vivo*, while overexpression of hsa_circ_0007843 effectively promoted colon cancer growth *in vivo.*

### Hsa_circ_0007843 Interacts with mIR-518c-5p, and mIR-518c-5p Interacts with MMP2

**Figure 3:**
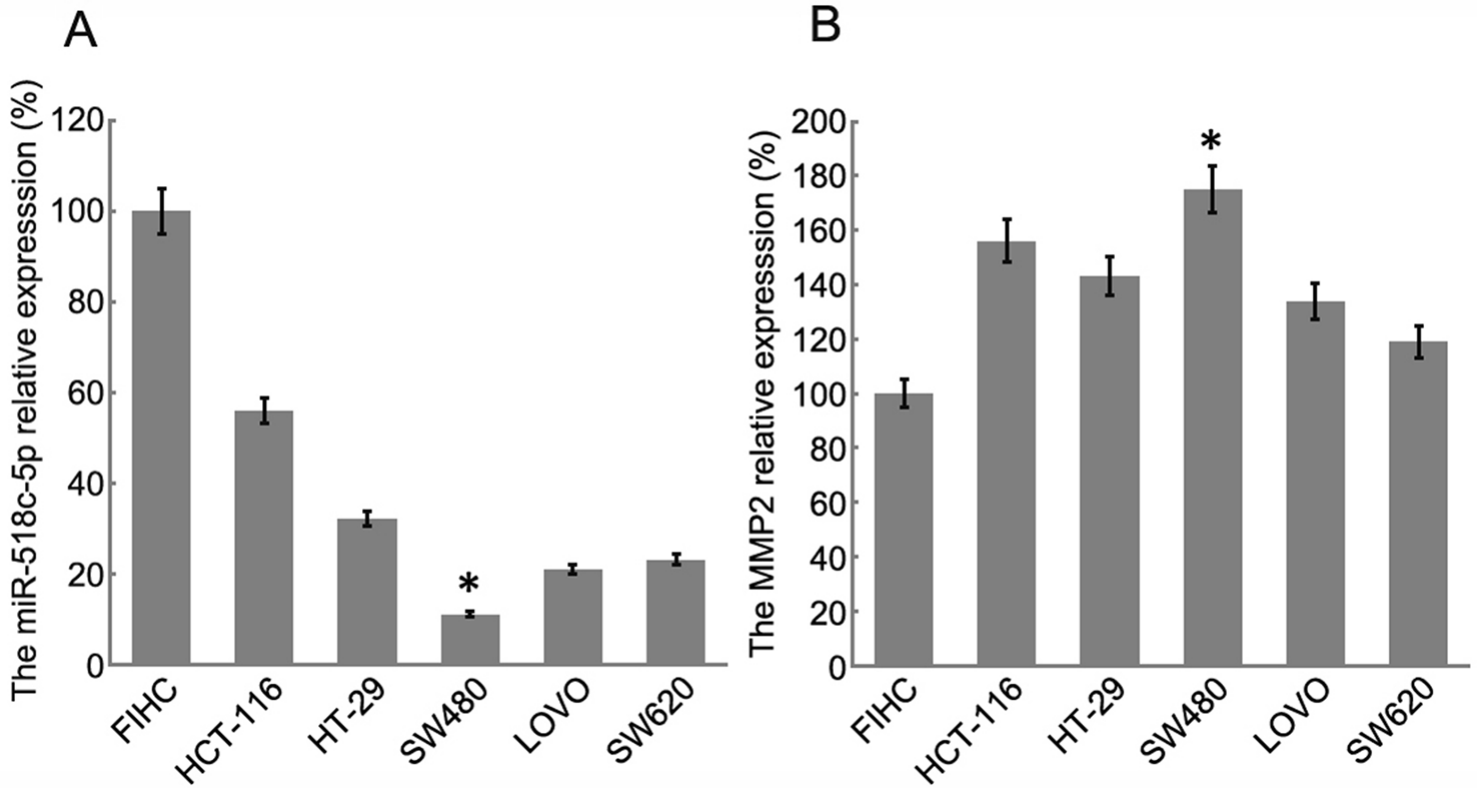
Relative expression of miR-518c-5p and MMP2 in colon cancer cell lines by RT-PCR. **(A)** Relative expression of miR-518c-5p; each bar represents the mean of 3 independent experiments. ^*^ *P* < 0.05. **(B)** Relative expression of MMP2; each bar represents the mean of 3 independent experiments. ^*^ *P* < 0.01.

**Figure 4:**
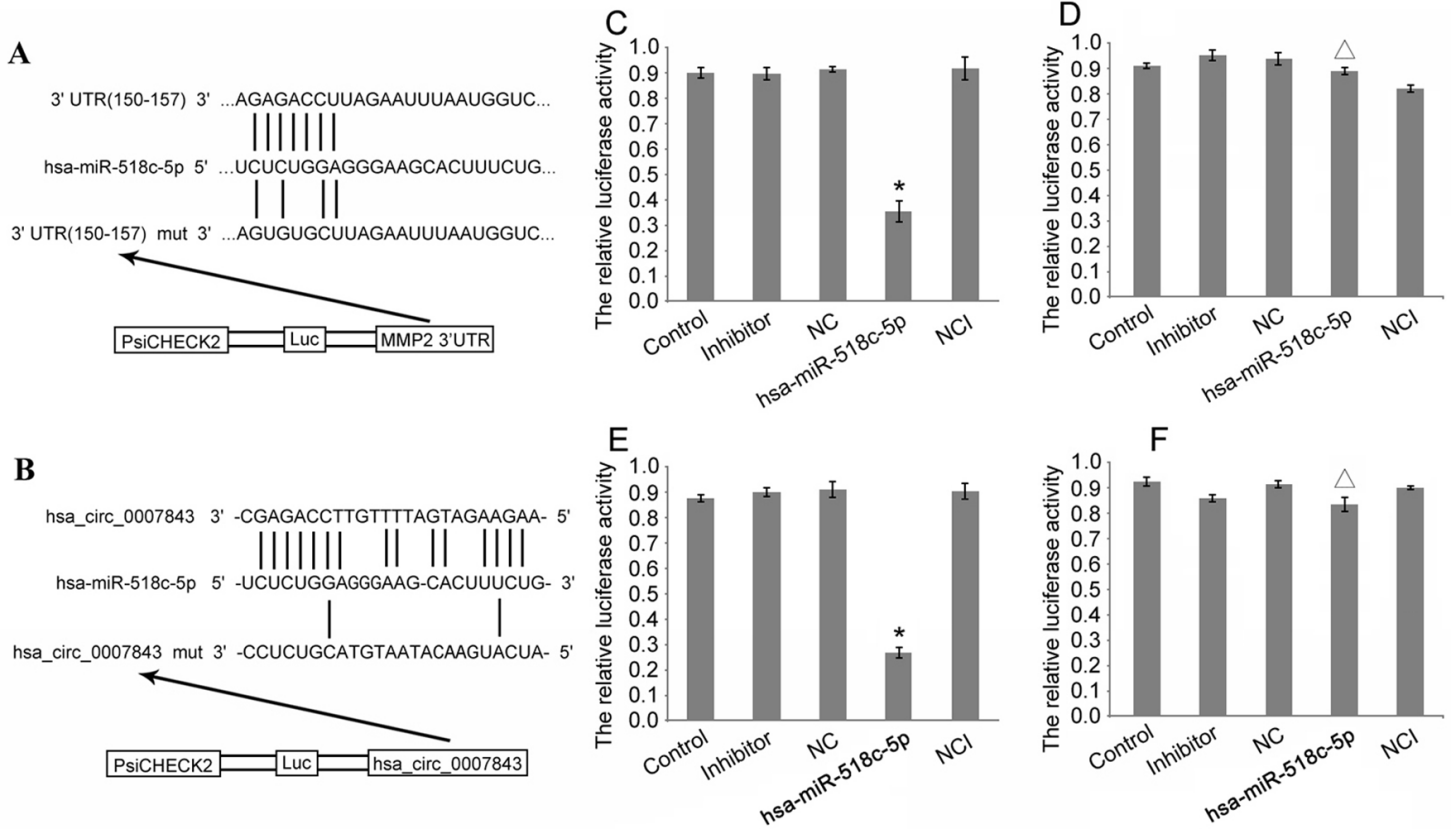
Construction of double fluorescent reporter gene vector to verify the interactions between hsa_circ_0007843, miR-518c-5p, and MMP2. **(A)** Target sequences for miR-518-5p in the 3′-UTR region of wild-type MMP2. **(B)** Target sequences for miR-518-5p on hsa_circ_0007843. **(C)** Comparison of luciferase activity in cells of different groups that were co-transfected with MMP2-3′UTR-WT; each bar represents the mean of 3 independent experiments. ^*^ *P* < 0.05. **(D)** Luciferase activity in cells of different groups that were co-transfected with MMP2-3′UTR-Mut; each bar represents the mean of 3 independent experiments. ^Δ^ *P* > 0.05. **(E)** Comparison of luciferase activity in cells of different groups that were co-transfected with hsa_circ_0007843-WT; each bar represents the mean of 3 independent experiments. ^*^ *P* < 0.05. **(F)** Luciferase activity in cells of different groups that were co-transfected with hsa_circ_0007843-Mut; each bar represents the mean of 3 independent experiments. ^Δ^ *P* > 0.05. Control group (psiCHECK-2-cirRNA/psiCHECK-2-mRNA, psiCHECK-2-cirRNA-mut/psiCHECK-2-mRNA-mut), inhibitor (inhibitor for miRNA + psiCHECK-2-cirRNA/psiCHECK-2-mRNA, psiCHECK-2-cirRNA-mut/psiCHECK-2-mRNA-mut), NC group (negative control sequence + psiCHECK-2-cirRNA/psiCHECK-2-mRNA or psiCHECK-2-cirRNA-mut/psiCHECK-2-mRNA-mut), NCI group (inhibitor for negative control + psiCHECK-2-cirRNA/psiCHECK-2-mRNA or psiCHECK-2-cirRNA-mut/psiCHECK-2-mRNA-mut sequence), hsa_miR-518c-5p group (hsa_miR-518c-5p mimics + psiCHECK-2-cirRNA/psiCHECK-2-mRNA or psiCHECK-2-cirRNA-mut/psiCHECK-2-mRNA-mut).

RT-PCR was conducted to determine the relative expression levels of miR-518c-5p and MMP2 in the colon cancer cell lines. The results indicated that MMP2 expression was upregulated in SW480 cells, whereas miR-518c-5p expression was downregulated in these cells (**Figure 3**). To further confirm the interaction between hsa_circ_0007843, miR-518c-5p, and MMP2, hsa_circ_0007843 wild-type (hsa_circ_0007843-WT), hsa_circ_0007843 mutant-type (hsa_circ_0007843-Mut), MMP2-3′ untranslated region (UTR) wild-type (MMP2-3′UTR-WT), and MMP2-3′UTR mutant type (MMP2-3′UTR-Mut) expression vectors were constructed and co-transfected with miR-518c-5p mimics into 293T cells, after which luciferase activity was examined. Our results indicated that luciferase activity was significantly decreased in 293T cells co-transfected with hsa_circ_0007843-WT, MMP2-3′UTR-WT, and miR-518c-5p mimics, suggesting that hsa_circ_0007843 might interact with miR-518c-5p, and miR-518c-5p might interact with MMP2. Furthermore, luciferase activity was not affected in 293T cells co-transfected with hsa_circ_0007843-Mut, MMP2-3′UTR-Mut, and miR-518c-5p mimics, suggesting that no interaction existed (**Figure 4**).

### Hsa_circ_0007843 Suppresses mIR-518c-5p Expression, and mIR-518c-5p Promotes MMP2 Expression

**Figure 5:**
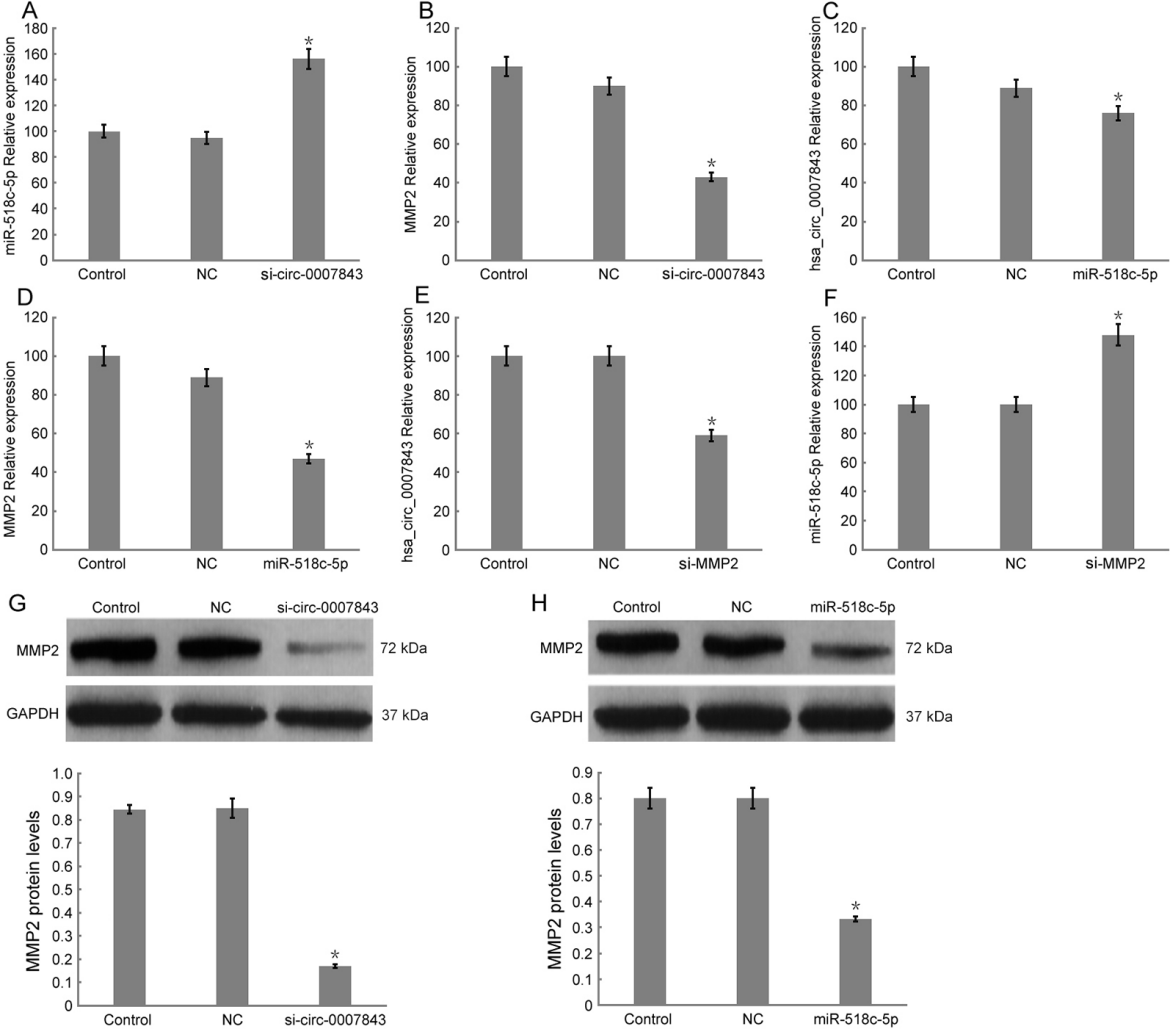
Relative expression levels of hsa_circ_0007843, miR-518c-5p, and MMP2 in SW480 cells by RT-PCR and western blotting. **(A)** Relative expression of miR-518c-5p upon downregulation of hsa_circ_0007843 expression; each bar represents the mean of 3 independent experiments. ^*^ *P* < 0.05. **(B)** Relative expression of MMP2 upon downregulation of hsa_circ_0007843 expression; each bar represents the mean of 3 independent experiments. ^*^ *P* < 0.01. **(C)** Relative expression of hsa_circ_0007843 upon overexpression of miR-518c-5p; each bar represents the mean of 3 independent experiments. ^*^ *P* < 0.05. **(D)** Relative expression of MMP2 upon overexpression of miR-518c-5p; each bar represents the mean of 3 independent experiments. ^*^ *P* < 0.01. **(E)** Relative expression of hsa_circ_0007843 upon downregulation of MMP2 expression; each bar represents the mean of 3 independent experiments. ^*^ *P* < 0.01. **(F)** Relative expression of miR-518c-5p upon downregulation of MMP2 expression; each bar represents the mean of 3 independent experiments. ^*^ *P* < 0.01. **(G)** Corresponding expression of MMP2 protein upon downregulation of hsa_circ_0007843 expression. **(H)** Corresponding expression of MMP2 protein upon overexpression of miR-518c-5p. Control group (no infection), si_hsa_circ_0007843 group (infected with lentiviral vector expressing hsa_circ_0007843 siRNA), hsa_circ_0007843 group (infected with lentiviral vector expressing hsa_circ_0007843), NC group (infected with lentiviral vector expressing negative control sequence), and miR-518c-5p group (infected with lentiviral vector overexpressing miR-518c-5p).

In order to elucidate the regulatory mechanism involving hsa_circ_0007843, miR-518c-5p, and MMP2, we downregulated hsa_circ_0007843 expression in SW480 cells and found that miR-518c-5p expression was upregulated, while MMP2 expression was downregulated (**Figures 5A, B, G**). At 48 h after infection of SW480 cells with a lentiviral vector overexpressing miR-518c-5p, the expression levels of hsa_circ_0007843 and MMP2 were decreased (**Figures 5C, D, H**). At 48 h after SW480 cells were infected with MMP2 siRNA, the expression level of hsa_circ_0007843 was decreased; however, miR-518c-5p expression was increased (**Figures 5E, F**). Because hsa_circ_0007843 could interact with miR-518c-5p, and because miR-518c-5p could interact with MMP2, we concluded that hsa_circ_0007843 interacted with miR-518c-5p to suppress its expression, while miR-518c-5p interacted with MMP2 to promote its expression.

## Discussion

In the present study, by using circRNA microarray and RT-PCR analyses, we identified four circRNAs (hsa_circ_0007843, hsa_circ_0010575, hsa_circ_0007331, and hsa_circ_0001615) that were significantly upregulated in colon cancer tissue. Among these four circRNAs, the expression of hsa_circ_0007843 was the most significantly increased and thereby selected for further study. Hsa_circ_0007843 is encoded by the ARHGAP32 (Rho GTPase activating protein 32arhgap32) gene, which is located at chr11:128993340-129034322 (http://www.circbase.org/) and has a role in the development of breast cancer (Ina et al., 2012). Our results showed that hsa_circ_0007843 overexpression could promote SW480 cell invasion and migration, whereas the downregulation of hsa_circ_0007843 expression could suppress SW480 cell invasion and migration. These results suggest that hsa_circ_0007843 plays a role in colon cancer development and could be considered as a potential biomarker for the early diagnosis of colon cancer. A previous study demonstrated that the expression of circRNAs is highly stable and shows tissue and developmental stage specificity (Li et al., 2015). Moreover, circRNAs are considered to be potential biomarkers for cancer diagnosis.

Li et al. reported that hsa_circ_002059 expression was significantly decreased in gastric cancer tissue, and its preoperative and post-operative levels in plasma were significantly different, which could be affected by distant metastasis, tumor-node-metastasis staging, sex, and age (Li et al., 2015). A total of 698 circRNAs were shown to be dysregulated in laryngeal carcinoma, with hsa_circ_100855 having the most significant increase in expression (Xuan et al., 2016). Hsa_circ_0001649 expression was found to be downregulated in hepatocellular carcinoma, and was considered to be a potential biomarker for the diagnosis and treatment of hepatocellular carcinoma (Qin et al., 2016). Wang et al. demonstrated that the expression of hsa_circ_001988 was decreased in colon cancer, which might be associated with the proliferation and eosinophilic invasion of colon cancer cells (Wang et al., 2015). Thus, hsa_circ_0007843 could play important roles in the pathogenesis and development of colon cancer.

An increasing number of studies have shown that some circRNAs have specific binding sites for miRNAs, and miRNAs can suppress the degradation of the corresponding mRNAs by competitively binding to endogenous circRNAs (Hansen et al., 2013). In the present study, we demonstrated that the expression levels of hsa_circ_0007843 and MMP2 were upregulated in SW480 cells, whereas miR-518c-5p expression was downregulated in these cells. These results suggested that hsa_circ_0007843 acted as an miRNA sponge to regulate MMP2 expression by removing the inhibitory effect of miR-518c-5p on MMP2 gene translation. Bioinformatics analysis and a luciferase reporter assay revealed that the 3′UTRs of hsa_circ_0007843 and MMP2 share identical miR-518c-5p response elements and could therefore bind competitively to miR-518c-5p. Using RT-PCR and western blotting analyses, we demonstrated that hsa_circ_0007843 suppressed miR-518c-5p expression, while miR-518c-5p promoted MMP2 expression. MMP2 is considered to be fundamental for the degradation of the extracellular matrix. Its expression level was found to be clearly upregulated in colon cancer, which might contribute to colon cancer cell invasion and migration (Schwegmann et al., 2016; Hsu et al., 2017). Hsa_circ_000984 acts as a competing endogenous RNA (ceRNA) by competitively binding to miR-106b and effectively upregulating CDK6 expression, thereby inducing a series of malignant phenotypes of tumor cells (Xu et al., 2017). In addition, hsa_circ_0055625 increases the growth of colon cancer cells by sponging miR-106b-5p (Zhang et al., 2019). CircCCDC66 expression is elevated in polyps and colon cancer and is associated with poor prognosis; circCCDC66 controls multiple pathological processes, including cell proliferation, migration, invasion, and anchorage-independent growth (Hsiao et al., 2017).

**Figure 6:**
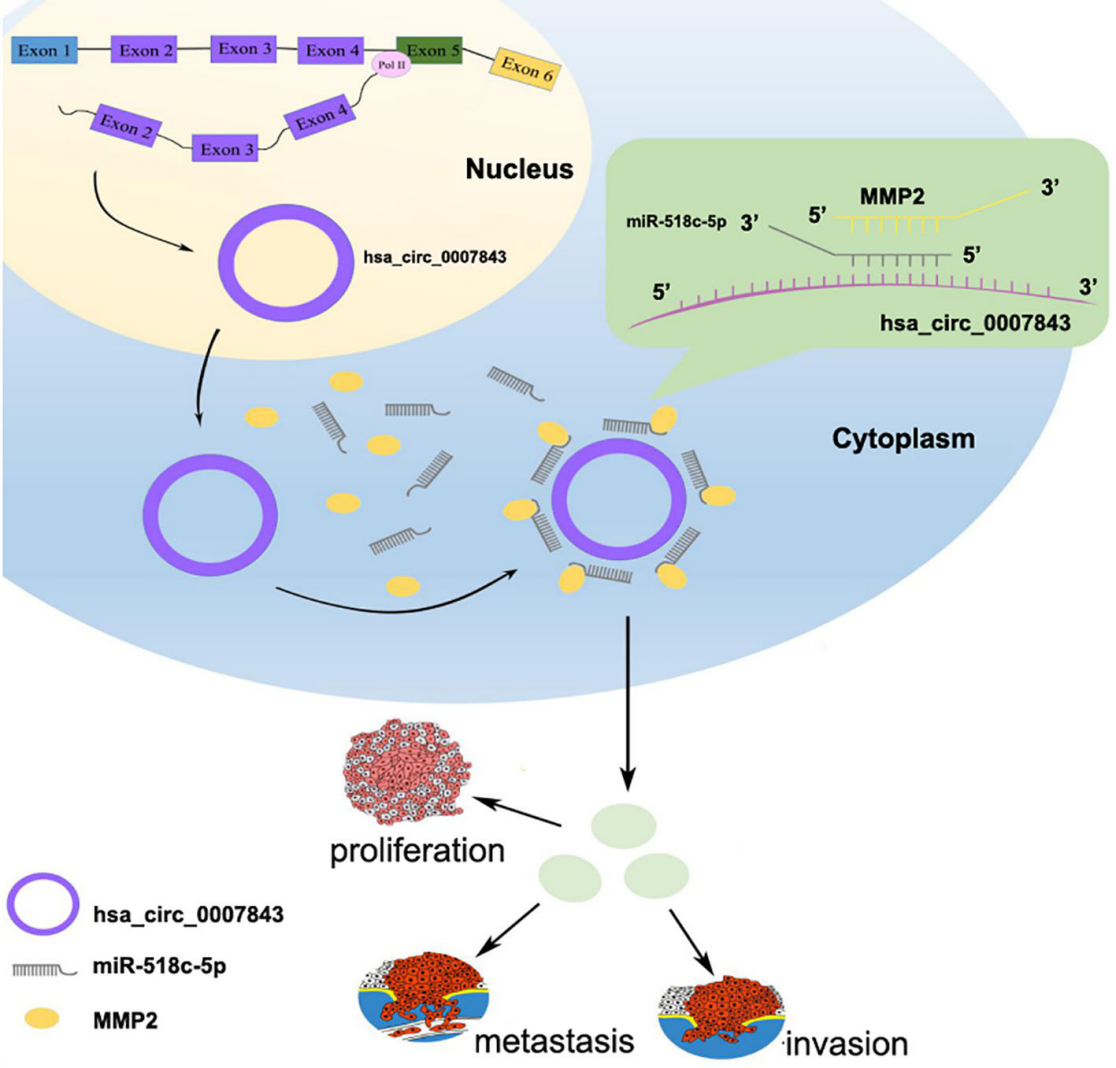
Hsa_circ_0007843/miR-518c-5p/MMP2 regulation axis effects on invasion and migration of SW480 cells.

In conclusion, our results suggest that hsa_circ_0007843, along with oncogenic characteristics, might be a potential biomarker for the diagnosis and treatment of colon cancer. Hsa_circ_0007843 overexpression promoted SW480 cell invasion and migration, whereas its downregulation suppressed SW480 cell invasion and migration. Hsa_circ_0007843 suppressed miR-518c-5p expression, while miR-518c-5p promoted MMP2 expression. Therefore, hsa_circ_0007843 acted as an miRNA sponge to regulate MMP2 expression by removing the inhibitory effect of miR-518c-5p on MMP2 gene translation, which further affected the invasion and migration of SW480 cells (**Figure 6**). Our findings suggested that the hsa_circ_0007843/miR-518c-5p/MMP2 regulation axis might play a critical role in the progression and development of colon cancer. The ceRNA network and pathway involved might be novel clinical markers and therapeutic targets for colon cancer patients.

## Data Availability Statement

The raw data supporting the conclusions of this article will be made available by the authors.

## Ethics Statement

The studies involving human participants were reviewed and approved by archives of the Central Hospital of Panyu District. The patients/participants provided their written informed consent to participate in this study. The animal study was reviewed and approved by JiNan University.

## Author Contributions

JH, ZH, YBL, MH and JBZ performed the experiments. JJ, JGL and LZ analyzed the data. JH and ZH wrote the manuscript. YGL and JDZ designed the study and revised the manuscript. WC and MH provided the reagents. All authors read and approved the final manuscript.

## Funding

This work was supported by grants from the Medical and Health Science and Technology Project of Panyu District, Guangzhou (No. 2017-Z04-18;2018-Z04-59;2019-Z04-02), Science and Technology Planning Project of Guangdong Province (No. 2017ZC0372), Guangzhou Health and Family Planning Commission Program (No. 20181A011118; 20192A011027; No. 20191A011119), Project of Guangdong Administration of Traditional Chinese Medicine (No. 20192073), Natural Science Foundation of Guangdong Province (No. 2018A0303130191), and Guangzhou Science and Technology Plan Project (No. 201904010044).

## Conflict of Interest

The authors declare that the research was conducted in the absence of any commercial or financial relationships that could be construed as a potential conflict of interest.

## Abbreviations

ceRNA, competing endogenous RNA; circRNA, circular RNA; miRNA, microRNA; MMP2, metalloproteinase 2; NC, negative control; NCI, negative control inhibitor; RT-PCR, reverse transcription polymerase chain reaction; UTR, untranslated region.
